# COVID-19-Associated Mucormycosis (CAM): An Updated Evidence Mapping

**DOI:** 10.3390/ijerph181910340

**Published:** 2021-09-30

**Authors:** Salman Hussain, Harveen Baxi, Abanoub Riad, Jitka Klugarová, Andrea Pokorná, Simona Slezáková, Radim Líčeník, Abul Kalam Najmi, Miloslav Klugar

**Affiliations:** 1 Czech National Centre for Evidence-Based Healthcare and Knowledge Translation (Cochrane Czech Republic, Czech EBHC: JBI Centre of Excellence, Masaryk University GRADE Centre), Institute of Biostatistics and Analyses, Faculty of Medicine, Masaryk University, Kamenice 5, 62500 Brno, Czech Republic; abanoub.riad@med.muni.cz (A.R.); klugarova@med.muni.cz (J.K.); apokorna@med.muni.cz (A.P.); simona.slezakova@med.muni.cz (S.S.); radim.licenik@gmail.com (R.L.); 2 Independent Researcher, New Delhi 110062, India; harveen94baxi@gmail.com; 3 Department of Public Health, Faculty of Medicine, Masaryk University, Kamenice 5, 62500 Brno, Czech Republic; 4 Department of Nursing and Midwifery, Faculty of Medicine, Masaryk University, Kamenice 5, 62500 Brno, Czech Republic; 5 Department of Pharmacology, School of Pharmaceutical Education and Research, Jamia Hamdard, New Delhi 110062, India; aknajmi@jamiahamdard.ac.in

**Keywords:** COVID-19, diabetes, epidemiology, evidence, mortality, mucormycosis, mycoses, public health

## Abstract

Mucormycosis, a serious and rare fungal infection, has recently been reported in COVID-19 patients worldwide. This study aims to map all the emerging evidence on the COVID-19-associated mucormycosis (CAM) with a special focus on clinical presentation, treatment modalities, and patient outcomes. An extensive literature search was performed in MEDLINE (Ovid), Embase (Ovid), Cochrane COVID-19 Study Register, and WHO COVID-19 database till 9 June 2021. The primary outcome was to summarize the clinical presentation, treatment modalities, and patient outcomes of CAM. Data were summarized using descriptive statistics and presented in tabular form. This evidence mapping was based on a total of 167 CAM patients with a mean age of 51 ± 14.62 years, and 56.28% of them were male. Diabetes mellitus (73.65% (n = 123)), hypertension (22.75% (n = 38)), and renal failure (10.77% (n = 18)) were the most common co-morbidities among CAM patients. The most common symptoms observed in CAM patients were facial pain, ptosis, proptosis, visual acuity, and vision loss. Survival was higher in patients who underwent both medical and surgical management (64.96%). Overall mortality among CAM patients was found to be 38.32%. In conclusion, this study found a high incidence of CAM with a high mortality rate. Optimal glycemic control and early identification of mucormycosis should be the priority to reduce the morbidity and mortality related to CAM.

## 1. Introduction

The coronavirus disease (COVID-19) outbreak caused by severe acute respiratory syndrome coronavirus 2 (SARS-CoV-2) has infected more than 228 million people globally, with about 4.7 million deaths as of 21 September 2021 [1]. The novel COVID-19 strains that have emerged this year are more severe variants of the disease and have resulted in higher intensive care unit (ICU) admissions, need for mechanical ventilation, and mortality [2,3]. This, consequently, has increased the burden on healthcare systems globally [4].

COVID-19 patients often have several comorbidities, including diabetes [5]. Ample evidence has found patients with comorbidities to be at higher risk of ICU admissions and mortality [5,6,7]. Study findings by Liu et al. from Wuhan Union Hospital found a more intense level of lymphocytopenia and cytokine storm in patients with severe COVID-19 compared to that in patients with mild disease [8]. Despite the colossal impact of this pandemic gripping the world, there are limited treatment options for it. COVID-19 patients in severe or critical stages (admitted to ICUs) are prescribed high doses of steroids as a life-saving measure [9]. Steroids suppress the immune system (decrease in CD4 + T and CD8 + T cells) to fight against the inflammation caused by the virus, thereby creating a favorable environment for other opportunistic infections [9,10]. This can make the immunocompromised COVID-19 patients more susceptible to a range of viral, bacterial, fungal, and other microbial co-infections [11]. Multiple studies have confirmed that patients with severe COVID-19 admitted to ICUs have a high occurrence of secondary infections and relatively infrequent bacterial co-infection [12,13,14].

Mucormycosis, a serious and rare fungal infection, has occurred concurrently in COVID-19 patients globally [15]. COVID-19-associated mucormycosis (CAM) notably created havoc in the second wave of COVID-19 in India. Mucormycosis, also known as black fungus, is an invasive fungal infection most commonly caused by species of the genus *Rhizopus* [16]. Other species causing this fungal infection include those belonging to the genera *Apophysomyces*, *Absidia*, *Mucor*, and others. Amongst the various types of mucormycosis, rhino-orbital-cerebral is the most common one [17]. Risk factors associated with the development of fungal infection among COVID-19 patients include diabetes, neutropenia, hematological malignancy, stem cell transplant recipients, patients receiving corticosteroid treatment, and individuals in the immunocompromised state [18,19]. Mucormycosis is associated with a high risk of all-cause mortality (54%), with mortality depending on body site infected, fungus type, and the patient’s overall condition [20].

This deadly fungal infection is clinically challenging and expensive to treat and puts a high toll on public health and a humanistic and economic burden on individuals and healthcare systems [21,22]. Low- and middle-income countries such as India witnessed a massive number of CAM cases in the second wave of COVID-19, leading to a collapse of the health system in the midst of the pandemic. The Indian government (state governments) declared mucormycosis as an outbreak in May 2021 [23]. Evidence from previous published studies was based on fewer cases and limited information [24,25].

Presently, more detailed evidence on the clinical presentation, treatment modalities, and patient outcomes is required. The preliminary search for mapping existing evidence was performed on 25 May 2021, in Epistemonikos, the international prospective register of systematic reviews (PROSPERO), Open Science Framework (OSF), Cochrane Library, and Jonna Briggs Institute (JBI) Evidence Synthesis, and no previous evidence mapping was identified. Therefore, we conducted this study with an objective to map all the emerging evidence on the CAM with a particular focus on each minute detail of clinical presentation, treatment modalities, and patient outcomes.

## 2. Materials and Methods

The proposed study was developed by adhering to the JBI methodology for evidence mapping and is reported as per the Preferred Reporting Items for Systematic Reviews and Meta-analyses for Scoping Reviews (PRISMA-ScR) [26,27]. Compliance with the PRISMA-ScR is presented in Supplementary Table S1.

Furthermore, this review was conducted by adhering to our protocol registered prospectively at OSF with an identification number (osf.io/438sm) and published as a preprint at the Preprint Server for Health Sciences (medRxiv) [28]. There were slight deviations from the protocol; firstly, the critical appraisal was skipped as it is not mandatory as per the JBI guidelines. The second deviation was the inclusion of suspected COVID-19 cases with confirmed mucormycosis, as patients developed mucormycosis after recovery from COVID-19.

### 2.1. Eligibility Criteria

#### 2.1.1. PCC Elements

According to the JBI reviewer’s manual, the following PCC (Population, Concept, and Context) elements were used for this review.

(a) Participants: patients with confirmed COVID-19 (RT-PCR) and mucormycosis (either histologically or microbiologically confirmed) based on the definition of Centers for Disease Control and Prevention were included in the study. We also included studies with suspected COVID-19 patients (based on the included studies assessment) who had confirmed mucormycosis.
(b) Concept and context: this review included all studies that described the clinical presentation, treatment modalities, and patient outcomes of CAM.

#### 2.1.2. Types of Sources

We included analytical observational studies (cohort, case–control) and descriptive observational studies (case report, case series, cross-sectional).

#### 2.1.3. Exclusion Criteria

(a) Non-English language studies;
(b) studies with no confirmed mucormycosis; and
(c) systematic reviews, narrative reviews, editorials, opinions, and study protocols were excluded.

### 2.2. Information Sources and Search Strategy

A three-step search strategy was utilized to identify published, unpublished, or ongoing studies with no language restrictions. An initial limited search was undertaken in MEDLINE (Ovid), followed by analyzing the text words in the title and abstract and the index terms assigned to the articles. Slightly modified Ovid Expert Searches for COVID-19 were combined with keywords and index terms related to mucormycosis to perform the searches in MEDLINE (Ovid) [29] and Embase (Ovid) [30] (Appendix A).

On 9 June 2021, we conducted a second search in MEDLINE (Ovid), Embase (Ovid), Cochrane COVID-19 Study Register, and the World Health Organization (WHO) COVID-19 database.

Complete search strategies are presented in Supplementary Table S2 for each database with their respective hits. Third, the manual search of reference lists of all included studies and relevant systematic reviews was screened for any potentially eligible studies. Citation tracking was also performed for all the included articles.

### 2.3. Selection Process

Two independent reviewers (S.H. and H.B.) screened all the retrieved articles against the eligibility criteria. We included all those articles describing the mucormycosis case (diagnosed either based on histopathology, culture, or stain) in COVID-19-positive patients.

**Figure 1:**
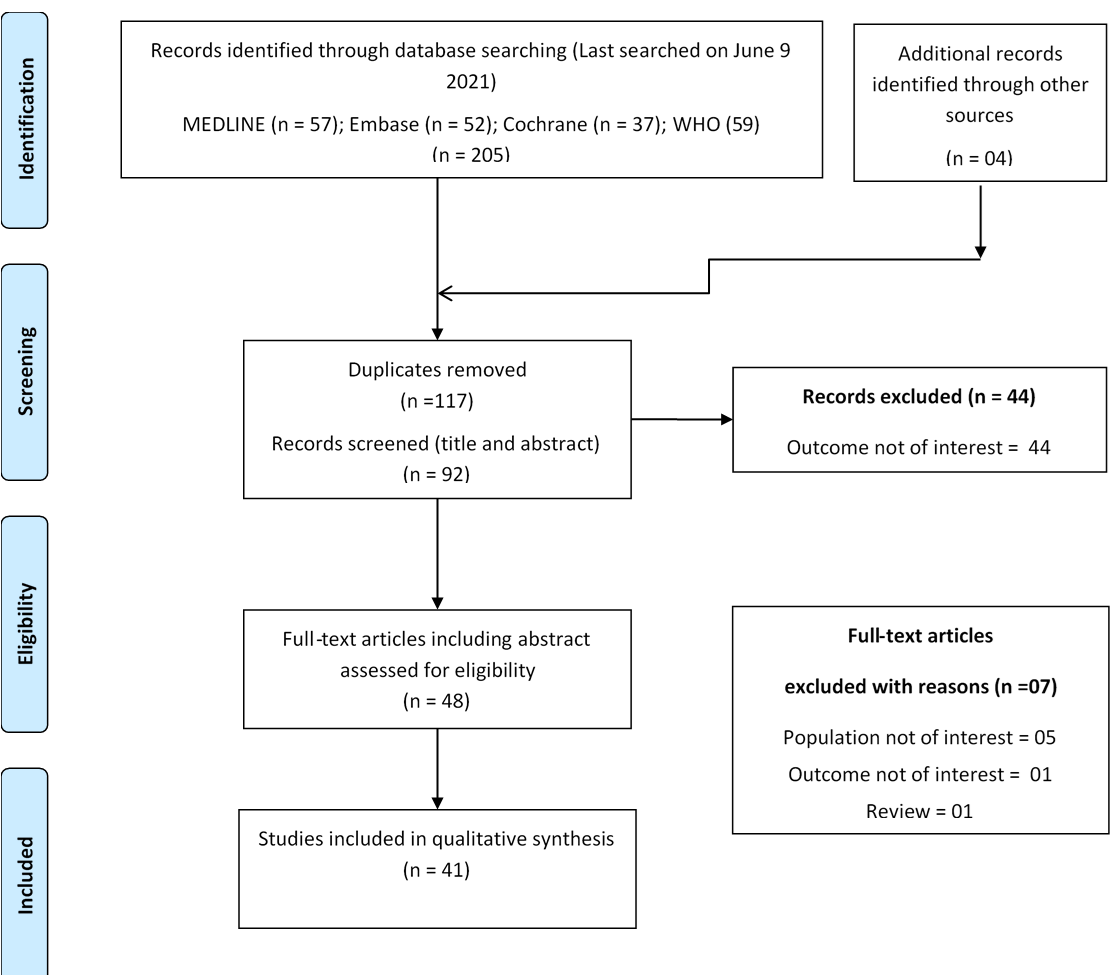
PRISMA flowchart showing study selection process.

In the initial screening phase, articles were selected based on the title and abstract scanning. In the second phase, full-text screening was performed for the final inclusion of articles. Any confusion regarding study inclusion was resolved by discussion with the third reviewer (M.K.). A detailed description of the study selection process is shown using the PRISMA flow diagram in Figure 1.

### 2.4. Data Extraction

Two reviewers (S.H. and H.B.) independently extracted the data in a pre-designed data extraction template. The following information was extracted from all the eligible studies qualified for inclusion: study author, year of publication, country, study design, demographic characteristics of the population (age and sex), sample size, comorbidities, treatment for COVID-19, symptoms of mucormycosis, diagnosis of mucormycosis, identification of fungal species, treatment for mucormycosis, and patient outcomes. The included studies are described using descriptive statistics and presented in a tabular form.

## 3. Results

A total of 209 articles were identified by searching the selected sources. After removing duplicates, only 92 articles were found to be unique. After the full-text screening, 37 studies [31,32,33,34,35,36,37,38,39,40,41,42,43,44,45,46,47,48,49,50,51,52,53,54,55,56,57,58,59,60,61,62,63,64,65,66,67] qualified for inclusion in this evidence mapping study. Four additional articles [68,69,70,71] were identified by hand search during bibliography screening and citation tracking. Finally, a total of 41 articles were included in this review [31,32,33,34,35,36,37,38,39,40,41,42,43,44,45,46,47,48,49,50,51,52,53,54,55,56,57,58,59,60,61,62,63,64,65,66,67,68,69,70,71]. Refer to Supplementary Table S3 for the list of articles excluded during full-text screening with reason.

### 3.1. Studies Characteristics

Out of 41 studies, the majority of studies (n = 15) were from India with 82 mucormycosis cases, 9 studies with 9 cases of mucormycosis were from the USA, while only 3 studies were from Iran but with 17 mucormycosis cases. Most of the included studies were case reports (n = 27) followed by case series (n = 9), and the rest were of other study designs. Diabetes mellitus (73.65% (n = 123)), hypertension (22.75% (n = 38)), and renal failure (10.77% (n = 18)) were the most common co-morbidities among CAM patients. Diabetic ketoacidosis was observed in one-tenth of the diabetic patients.

### 3.2. Clinical Presentation

This evidence mapping was based on a total of 167 CAM patients with a mean age of 51 ± 14.62 years, of which 56.28% of them were male. COVID-19 was confirmed through the RT-PCR test in approximately three-fourth (74%) of the included studies.

The majority of the patients (76.04%) were treated using steroids, while only 11.64% of patients were treated with remdesivir to manage COVID-19. Most patients who developed mucormycosis had severe (based on included studies’ categorization) or critical COVID-19 (defined based on ICU status/mechanical ventilation).

Twenty-nine (17.57%) patients had concurrent CAM, while the remaining patients were diagnosed with CAM after an average of 19.24 days. Mucormycosis was diagnosed using stain (24 studies), culture (26 studies), or histopathology (30 studies), and nine studies diagnosed mucormycosis using all three diagnostic techniques. The *Rhizopus* species were the most common fungal species infecting CAM patients (13.77%).

Facial pain, ptosis, proptosis, visual acuity, and vision loss were the most common symptoms observed in CAM patients. Rhino-orbital (16%) followed by rhino-orbital-cerebral (11.3%) mucormycosis was the most common form of mucormycosis found in CAM patients (Table 1).

### 3.3. Treatment Modalities and Outcomes

Liposomal amphotericin B in various doses (5 mg/kg/day) was the most commonly used drug for managing mucormycosis infection in 158 patients (35 studies). Adjunct surgery was performed on 142 patients, and surgical debridement was the most common surgical procedure performed. Only 23 CAM patients were managed without surgery, and most of them (18 CAM patients) died between 7 to 62 days after the diagnosis of mucormycosis.

Survival was higher in patients who underwent both medical and surgical management (64.96%) than in CAM patients who underwent medical management only (21.73%). Overall mortality among CAM patients in the included studies was 38.32% (n = 64). The patients died between 6 to 90 days after mucormycosis diagnosis (Table 2).

## 4. Discussion

To the best of our knowledge, this is the most comprehensive and up-to-date evidence mapping aimed to explore the published and unpublished evidence on the clinical presentation, treatment modalities, and patient outcomes of CAM. The current body of evidence was based on the 41 studies that met our inclusion criteria and discussed the association of COVID-19 with mucormycosis.

Mucormycosis is a rare opportunistic infection, and COVID-19 patients are at risk of developing mucormycosis because of pre-compromised immune systems. A growing body of evidence supports that comorbidities (diabetes, transplantation, malignancies) and medications (steroids) make the patients more vulnerable to CAM [5,6,7]. A recent case report found an invasive pulmonary mucormycosis case in a patient after a short course of steroids [72]. Likewise, Pan et al. found mucormycosis in a patient with AIDS receiving short-term systemic steroids [73]. In our study, we found that COVID-19 patients with comorbidities had a higher occurrence of mucormycosis.

Around 50% of CAM cases in our study were reported from India. A possible reason for this could be the deadly COVID-19 delta variant wave infecting around half a million people every day in recent months and a high prevalence of diabetes mellitus in CAM patients [74]. Diabetes mellitus is a predisposing factor for the development of mucormycosis [75,76]. The potential mechanism behind this could be the aggravation of the inflammatory state due to hyperglycemia and activation of antiviral immunity [77]. The risk of developing CAM increases significantly in patients with diabetic ketoacidosis, where Mucorales use free iron levels in the serum for pathogenesis [78].

In our study, the number of male mucormycosis patients was twice the number of female patients. These findings are aligned with a previously published study by Roden et al. [79] that found mucormycosis in 65% of male patients.

Rhino-orbital and rhino-orbital-cerebral were the most common forms of mucormycosis observed in this study. In both forms of infection, the fungus invades the nasal mucosa and orbital wall and leads to the occurrence of symptoms such as facial pain, vision loss, proptosis, apoptosis, and ophthalmoplegia [80,81]. CAM patients who underwent both surgical and medical management had a better survival rate than those with medical management alone. Published studies from different parts of the world have also found better outcomes in mucormycosis patients who underwent combined surgical and medical management [82,83]. However, despite the best management of CAM patients, the overall mortality was high, suggesting the need for the early identification of cases.

Our study findings suggest that clinical practitioners (intensivists and their teams) should be alerted about the increased possibility of CAM in critically ill COVID-19 patients; therefore, they should act proactively and monitor for potential fungal and bacterial co-infections and secondary infections among the COVID-19 cohorts, especially the immunocompromised and diabetic patients [84]. Moreover, these findings call drug regulators and health systems, especially in low- and lower-middle-income countries, to implement strict policies for steroid stewardship.

### 4.1. Limitations

Like every study, this evidence mapping has few limitations. Firstly, we could not differentiate the outcome based on glycemic-controlled status due to the lack of information on the glycosylated hemoglobin value of the CAM patients with diabetes in the included studies. Secondly, there was variability in the definition of severity of COVID-19 in the included studies. Lastly, limited information (fungal species identified, RT-PCR result) in a few included studies was also a drawback.

### 4.2. Strengths

The major strength of this review was a large number of exhaustive literature searches in major databases, a protocol-oriented approach, most up-to-date evidence with sound methodology, and the capture of each minute detail of 167 CAM patients to make this review a one-stop source of information for CAM.

## 5. Conclusions

This evidence mapping found a high incidence of CAM with a high mortality rate. Therefore, clinicians should cautiously use the steroids using the risk–benefit analysis approach. Optimal glycemic control and early identification of mucormycosis should be the priority to reduce the morbidity and mortality related to CAM.

## Figures and Tables

**Table 1:** Summary of study characteristics and anamnestic, diagnostic, and treatment features of COVID-19-associated mucormycosis (CAM) cases.

Study	Country	Design	n	Sex	Age (years)	COVID-19 Confirm.	COVID-19 Severity	Onset (days)	Comorbidities					COVID-19 Treatment		Clinical Features	Region	Diagnosis			Genus/Species
									DM	HTN	Asthma	CAD	Other	Steroids	Others			Stain	Cult.	Histo.	
Alekseyev et al. 2021 [31]	USA	Case report	1	M	41	RT-PCR	NR	NR	Yes	No	No	No	DKA	Yes (name NS)	HCQ	NS	NS	No	No	Yes	NS
Arana et al. 2021 [32]	Spain	Case report	1	M	62	RT-PCR	Severe (requiring non-invasive mechanical ventilation)	7	Yes	Yes	No	Yes	ESKD	Dexamethasone 6 mg daily for 10 days	Ceftriaxone, azithromycin	Fever, headache and left malar region swelling	Rhinosinusal	No	Yes	No	*Rhizopus/Rhizopus oryzae*
			1	M	48	RT-PCR	Moderate (FiO_2_: 28%)	21	No	Yes	No	No	ESKD	Prednisone 20 mg	HCQ, azithromycin, lopinavir/ritonavir, tocilizumab	Pain and increase in right limb diameter	Musculoskeletal	No	Yes	No	Lichtheimiaceae/*Lichtheimia ramosa*
Ashour et al. 2021 [33]	Egypt	Case series	6	M/F: 3/3	54.66	RT-PCR (2); NR (4)	Critical (n = 1) on ventilation; NR (n = 5)	Not clear	Yes (100%)	No	No	No	CKD (12.5%)	NR	NR	Ophthalmoplegia (66%), conjunctival chemosis (33%), eyelid edema (33%), facial edema (33%)	Rhino-orbital-cerebral (100%)	No	Yes	Yes	NS
Bayram et al. 2021 [34]	Turkey	Case series	11	M/F: 9/2	73.1 ± 7.7 years (range: 61–88 years)	Suspected	Severe (oxygen saturation <93% in room air)	14.4 ± 4.3 (range: 7–23 days)	Yes (73%)	Yes (64%)		Yes (18%)	Renal failure (45%)	Dexamethasone (100%)	NR	Proptosis (100%), ophthalmoplegia (64%), orbital pain (82%), conjunctival hyperemia or chemosis (82%), ptosis (64%), fixed and dilated pupil (64%), vision loss (64%), endophthalmitis (54.5%), and decreased vision (27%)	NR	Yes	Yes	Yes	NR
Bellanger et al. 2021 [35]	France	Case report	1	M	55	RT-PCR	Severe (ICU)	21	No	No	No	No	Follicular lymphoma, influenza B	NR	NR	Worsening of respiratory symptoms	NR	NR	Yes	Yes	*Rhizopus/Rhizopus microsporus*
Dallalzadeh et al. 2021 [36]	USA	Case report	1	M	48	RT-PCR	Critical (ICU, ventilation)	6	Yes	No	No	No	Ketoacidosis	Dexamethasone	CCP (COVID-19 convalescent plasma)	NR	Rhino-orbital	Yes	Yes	No	*Rhizopus/Rhizopus* species
El-Kohly et al. 2021 * [38]	Egypt	Cross-sectional	28	M/F: 19/17	52.92 ± 11.30	RT-PCR	Mixed (mild (n = 11), moderate (n = 13), severe (n = 12))	17.82 ± 2.97	Yes (27.8%)	Yes (17%)	Yes (8%)	No	CKD (8%)	Yes (name NS)	Antiviral, anticoagulant, and vitamins (name NS)	Headache and facial pain (75%), facial numbness (67%), ophthalmoplegia, and visual loss (64%), ophthalmoplegia (64%), diplopia (17%)	Sinonasal (100%), orbital (81%), cerebral (29%), and palatine (33%)	Yes	Yes	Yes	*Mucor*-species
Evert et al. 2020 [70]	Germany	Case series	2	F	52.5	RT-PCR	Critical (n = 2 on ventilation)	NR	No	No	No	No	Obesity, liver cirrhosis	Yes	NR	NR	NR	No	No	Yes	*Mucor*-species
Garg et al. 2021 [39]	India	Case report	1	M	55	RT-PCR	Severe (84% SpO_2_)	21	Yes	Yes	No	No	ESRD, Ischemic cardiomyopathy, venous thrombosis	Dexamethasone (6 mg, once a day for 14 days)	Remdesivir (200 mg on day 1 and 100 mg on days 2–5); supportive care	Cavitary pneumonia with pleural effusion	Pulmonary mucormycosis/cavitary pneumonia with pleural effusion	Yes	Yes	No	*Rhizopus/Rhizopus microsporus*
Hanley et al. 2020 (Autopsy) [40]	UK	Case series	1	M	22	RT-PCR	Critical (mechanical ventilation, vasopressor, ICU)	Concurrent	NR	NR	NR	NR	Frank necrotic- hemorrhagic pancreatitis; renal failure	NR	NR	NR	NR	Yes	No	Yes	NR
Johnson et al. 2021 [41]	USA	Case report	1	M	79	RT-PCR	Critical (ICU, ventilation)	19	Yes	Yes	No	No	Pulmonary aspergillosis	IV dexamethasone (6 mg daily for 10 days)	IV remdesivir (200 mg × 1, then 100 mg daily)	NR	NR	No	Yes (BAL culture)	Yes	*Rhizopus/Rhizopus arrhizus*
Junior et al. 2020 [37]	Brazil	Case report	1	M	86	Throat swab	Severe (ICU)	Concurrent	No	Yes	No	No	NR	Hydrocortisone	Oseltamivir	Mild abdominal tenderness	NR	Yes	No	Yes	NR
Kanwar et al. 2021 [42]	USA	Case report	1	M	56	RT-PCR	Severe	13	No	No	No	No	ESRD	Methylprednisolone	Tocilizumab	Necrotizing pneumonia with empyema	NR	Yes	Yes	No	*Rhizopus/Rhizopus azygosporus*
Karimi-Galougah et al. 2021 [43]	Iran	Case report	1	F	61	RT-PCR	NR	21	Yes	No	No	No	NR	Yes (name NS)	Remdesivir, interferon alpha	Hemifacial pain, proptosis, frozen eye, complete loss of vision, and fixed mydriasis	Rhino-orbital	NR	NR	Yes	NR
Khatri et al. 2021 [44]	USA	Case report	1	M	68	Suspected	Critical	90	Yes	Yes	No	Yes	Severe heart failure, obstructive sleep apnea; renal failure	Methylprednisolone/Prednisone (for gout)	CCP	Purplish skin discoloration with fluctuant swelling	Cutaneous	Yes	Yes	Yes	*Rhizopus/Rhizopus microsporus*
Khan et al. 2020 [71]	USA	Case report	1	F	44	RT-PCR	Critical (ICU, ventilation)	13	Yes	No	No	No	No	Methylprednisolone 30 mg IV twice a day	Remdesivir 100 mg IV daily	NR	Pulmonary mucormycosis	Yes	Yes	Yes	NS
Krishna et al. 2021 [45]	India	Case report	1	M	34	RT-PCR	Severe	NR	Yes	Yes	No	No	NR	NR	NR	Swelling pain over the first quadrant teeth	Sinonasal	No	No	Yes	NR
Krishna et al. 2021 [46]	UK	Case report (autopsy)	1	M	22	RT-PCR	Severe (mechanical ventilation)	Autopsy	No	No	No	No	No	Yes (name NS)	Meropenem and teicoplanin	Thrombo-emboli were seen in the lungs, brain, pharynx, nasal mucosa, and trachea	NR	Yes	No	No	Mucorales/NS
Maini et al. 2021 [47]	India	Case report	1	M	38	RT-PCR	Severe (ICU)	18	No	No	No	No	NO	Methylprednisolone (80 mg/day)	Inj. remdesivir IV with a loading dose of 200 mg, followed by 100 mg daily for 11 days.	Swelling and pain in the left eye	Rhino-orbital-cerebral	Yes	Yes	Yes	*Rhizopus/Rhizopus oryzae*
Mehta et al. 2020 [48]	India	Case report	1	M	60	RT-PCR	Critical (ICU, ventilation)	11	Yes	No	No	No	No	Methylprednisolone (40 mg twice daily) and dexamethasone (4 mg twice daily)	Oseltamivir (75 mg twice daily), later tocilizumab (400 mg)	Bilateral lid edema with right eye prominence, febrile, breathless, and hypoxic	Rhino-orbital-cerebral	Yes	Yes	Yes	Mucorales/unspecified
Mekonnen et al. 2021. [49]	USA	Case report	1	M	60	Suspected	Critical (mechanical ventilation, ICU)	4	Yes	Yes	Yes	No	AKI	Dexamethasone	CCP	Proptosis, erythema and edema of the eyelids, and conjunctival chemosis	Rhino-orbital	Yes	Yes	Yes	*Rhizopus/Rhizopus* species
Meshram et al. 2021 [50]	India	Case report (renal transplant recipients)	2	M	47; 25	Suspected	Mild	NR	Yes	No	No	No	No	NR	NR	Swelling over the face and black nasal discharge (50%); fever, cough, and black expectoration (50%)	Rhino-orbito-cerebral	No	Yes	Yes	No
Mishra et al. 2021 [68]	India	Case series	10	M/F: 9/1	55.8	Suspected	Mixed (mild (n = 3); moderate (n = 6); severe (n = 1))	NR	Yes (80%)	Yes (30%)	No	No	CKD (20%)	Yes (60%)	Remdesivir (50%)	Eye pain, facial pain and nasal block	NS	No	No	Yes	NS
Moorthy et al. 2021 [51]	India	Case series	17	M:15, F:2	54.6, 35–73 (mean, range)	RT-PCR	Not specified	Concurrent (n = 4)	Yes (82.73%)-14	No	No	No	No	Yes (100%)	NS	Orbital cellulitis, facial swelling, headache, proptosis, oedema of the extraocular muscles, ophthalmoplegia	Sinusitis alone (n = 3), rhino- orbital (n = 6), rhino-orbital- cerebral (n = 5), rhino-cerebral (n = 3)	Yes	No	Yes	Mucorales/unspecified
Nehara et al. 2021 [52]	India	Case series	5	M/F: 1/4	62.2 Average age	RT-PCR	NR	NR	Yes (100%)	Yes (40%)	No	No	Yes (20%)	Dexamethasone	Oxygen supplementation, intravenous meropenem, remdesivir (40%), subcutaneous enoxaparin, tablet azithromycin, basal-bolus insulin, and supportive care	Severe headache, diminished vision, chemosis, mild proptosis, complete ophthalmoplegia, blackish discharge from the nasal cavity, and black crust on the hard palate	Rhinocerebral	Yes	Yes	Yes	*Rhizopus/Rhizopus arrhizus*
Pakdel et al. 2021 [53]	Iran	Cross-sectional	15	M: 10; F: 5	Median age: 52 (14–71)	RT-PCR	Severe (34%)	Median: 7 (1–37)	Yes (87%)	Yes (46%)	Yes (13%)	No	Ketoacidosis (6%)	Dexamethsaone (46%)	Yes (7%)	Unilateral periorbital pain and edema (73%), eyelid ptosis (73%), acute vision loss (73%), proptosis (73%), unilateral facial edema (60%), cranial nerve palsy (60%), headache (33%), fever (27%), nasal blockage (13%), and ear pain (7%)	Mixed (rhinorbital (47%); sino-orbital (33%), isolated orbital movement (13%), and others)	Yes	No	Yes	NS
Pasero et al. 2020 [54]	Italy (renal transplant)	Case report	1	M	66	RT-PCR	Critical (ICU)	14	No	Yes	No	No	Renal failure	No	HCQS, lopinavir, ritonavir	NS	NS	Yes	Yes	No	*Rhizopus/Rhizopus* species
Pauli et al. 2021 [55]	Brazil	Case report	1	F	50	Suspected	Mild	8	Yes	No	No	No	No	Hydrocortisone	NR	Deep ulcerated lesion located at the center of the hard palate	Palatal ulcer	Yes	No	Yes	Mucorales/unspecified)
Placik et al. 2020 [56]	USA	Case report	1	M	49	RT-PCR	Critical	14	No	No	No	No	No	Dexamethasone	Remdesivir, tocilizumab	Necrotizing pneumonia with bronchopleural fistula	NS	Yes	Yes	Yes	*Rhizopus/Rhizopus* species
Rabagliati et al. 2021 [57]	Chile	Retrospective cohort study	1	M	55	Suspected	Critical (ICU)	Not specified	Yes	Yes	No	Yes	Atrial fibrillation	812 mg prednisone equivalent	No	NS	NS	No	Yes	No	*Rhizopus/Rhizopus microsporus*
Rao et al. 2021 [58]	India	Case report	1	M	66	Suspected	NR	NR	No	No	No	No	No	Systematic steroids	NR	Complete left ptosis and proptosis, chemosis and fixed dilated left pupil, and absence of left ocular movements in all directions of gaze, vision loss in the left eye	Rhino-orbito-cerebral	No	Yes	No	Fungal hyphae
Ravani et al. 2021 [59]	India	Retrospective cohort	18	NR	NR	RT-PCR	NR	60	Yes (100%)	NR	NR	NR	NR	Dexamethasone	NR	Diminution of vision (<6/60 in 81% of patients) and ophthalmoplegia (77%), orbital cellulitis (61%), pansinusitis (77%)	NR	No	No	Yes	NS
Revannavar et al. 2021 [60]	India	Case report	1	F	NR	RT-PCR	Mild	Not specified	Yes	No	No	No	No	NR	NR	Left-sided facial pain, complete ptosis and fever, tenderness of all sinuses on left side, ophthalmoplegia (left eye), left eye visual acuity	NS	No	Yes	Yes	*Rhizopus/Rhizopus* species
Saldanha et al. 2021 [61]	India	Case report	1	F	32	RT-PCR	Not specified	Concurrent	Yes	No	No	No	No	NR	NR	Left eye complete ptosis and left facial pain, visual acuity (left eye)	NS	No	Yes	Yes	NS
Sarkar et al. 2021 [62]	India	Case series	6	M:4, F:2	44	RT-PCR	Critical (n = 6)	Concurrent	Yes (100%)	No	No	No	Ketoacidosis (33%)	Dexamethasone	Remdesivir (84%)	Visual acquity (100%)	Rhino-orbital (n = 5), rhino- orbital-cerebral (n = 1)	Yes	Yes	No	*Rhizopus* (n = 4), Mucorales (n = 2)
Satish et al. 2021 [69]	India	Case series	11	NR	NR	RT-PCR	Mixed (mild (n = 2); moderate (n = 3); severe (n = 4); asymptomatic (n = 2)	NR	Yes (100%)	No	No	No	No	NR	NR	NR	NS	Yes	No	No	NR
Sen et al. 2021 [63]	India	Retrospective cohort	6 (5 patients post covid-19 recoved)	M	60.5 ± 12 (range 46.2 to 73.9) years	RT-PCR	Severe	NR	Yes (100%)	Yes (50%)	No	Yes (16.6%)	Diabetic ketoacidosis (50%)	Intravenous methylprednisolone/dexamethasone/oral prednisolone (84%)	No	Pain, redness, and periocular swelling, drooping of eyelids, limitation of ocular movements, and painful loss of vision	Rhino-orbital-cerebral	No	Yes	Yes	Mucorales/unspecified
Veisi et al. 2021 [64]	Iran	Case report	1	F	40	RT-PCR	Mild	NR	No	No	No	No	No	Dexamethasone (8 mg/day)	Remdesivir 200 mg on day 1 followed by 100 mg daily for 4 days, and IV levofloxacin (500 mg/day),	Bilateral visual loss, periorbital pain, and visual acuity	Rhino-orbito-cerebral	NR	NR	Yes	NR
			1	M	54	RT-PCR	NR	NR	Yes	No	No	No		Dexamethasone (8 mg/day)	Remdesivir 200 mg on day 1 followed by 100 mg daily for 4 days, IV levofloxacin (500 mg/day)	Left orbital pain and periorbital swelling together with progressive vision loss	Rhino-orbital	NR	NR	Yes	NR
Waizel-Haiat et al. 2021 [65]	Mexico	Case report	1	F	24	RT-PCR	Critical (ICU)	Concurrent	Yes	No	No	No	Ketoacidosis, renal failure	NA	NA	Left lid swelling and maxillary hypoesthesia, left hyperemic conjunctiva, and an opaque cornea	Rhino-orbital	Yes	Yes	No	*Lichtheimia* (*Absidia*) species
Werthman-Ehrenreich et al. 2021 [66]	USA	Case report	1	F	33	Suspected	Severe (ICU)	Concurrent	Yes	Yes	Yes	No	Ketoacidosis, renal failure	No	Remdesivir, CCP	Eye ptosis	Rhino-orbital-cerebral	Yes	Yes	No	Mucorales/unspecified
Zurl et al. 2021 [67]	Austria	Case report	1	M	53	RT-PCR	Critical (ICU)	Concurrent	No	No	No	No	Myelodysplastic syndromes, acute myeloid leukemia	Prednisolone	Tocilizumab	NR	Fungal pneumonia with effusion	Yes	No	Yes	*Rhizopus*/*Rhizopus microsporus*

CAD: coronary artery disease; DM: diabetes mellitus; ESRD: end-stage renal disease; F: female; HCQs: hydroxychloroquine; HTN: hypertension; ICU: intensive care unit; IV: intravenous; M: male; NR: not reported; NS: not specified; RT-PCR: reverse-transcriptase polymerase chain reaction; USA: United States of America. * No separate outcomes reported for mucormycosis (n = 28).

**Table 2:** Treatment details and patient outcomes.

Study (Author, Year)	Country	Treatment		Patient Outcome *
		Medical Management	Surgical Management	
Alekseyev et al. 2021 [31]	USA	NR	Yes	Lived
Arana et al. 2021 [32]	Spain	Amphotericin B (LAmB 5 mg/kg/day), isavuconazole, and subsequently posaconazole	Yes (surgical debridement)	Lived
		Amphotericin B (LAmB 5 mg/kg/day) together with isavuconazole 200 mg/8 h for 24 days	Yes (surgical debridement)	Lived
Ashour et al. 2021 [33]	Egypt	Amphotericin B	Yes (surgical debridement (n = 4))	Lived (67%), Died (33%)
Bayram et al. 2021 [34]	Turkey	Amphotericin B, voriconazole	Yes (all patients: endoscopic sinus surgery with extensive debridement)	Lived (36%), Died (64%)
Bellanger et al. 2021 [35]	France	Amphotericin B (LAmB 5 mg/kg/day)	No	Died
Dallalzadeh et al. 2021 [36]	USA	AMB/isavuconazole	No	Died
El-Kohly et al. 2021 * [38]	Egypt	Amphotericin B; voriconazole; posaconazole	Yes (endoscopic debridement (n = 27))	Lived (64%), Died (36%)
Evert et al. 2020 [70]	Germany	NR	NR	Died (100%)
Garg et al. 2021 [39]	India	Amphotericin B (LAmB 5 mg/kg/day)	No	Lived
Hanley et al. 2020 [40]	UK	NR	NR	Died
Johnson et al. 2021 [41]	USA	Amphotericin B (LAmB 400 mg daily)	Yes (no tracheostomy, and percutaneous endoscopic gastrostomy)	Lived
Junior et al. 2020 [37]	Brazil	No	NR	Died
Kanwar et al. 2021 [42]	USA	Amphotericin B (LAmB 5 mg/kg/day)	Yes (robotic decortication surgery)	Died
Karimi-Galougah et al. 2021 [43]	Iran	Yes (not specified)	Yes (endonasal endoscopic debridement of necrotic tissue, right eye exenteration)	Lived
Khatri et al. 2021 [44]	USA	Amphotericin B + posaconazole	Yes (thoracic cavity debridement)	Died
Khan et al. 2020 [71]	USA	Amphotericin B (5 mg/kg/day)	No	Died
Krishna et al. 2021 [45]	India	Amphotericin B (LAmB 5 mg/kg/day)	Yes (surgical resection)	Lived
Krishna et al. 2021 [46]	UK	Caspofungin	No	Died
Maini et al. 2021 [47]	India	Amphotericin B 300 mg/day, tobramycin and fluconazole	Yes (debridement)	Lived
Mehta et al. 2020 [48]	India	Amphotericin B	No	Died
Mekonnen et al. 2021. [49]	USA	Amphotericin B (LAmB) + caspofungin/posaconazole	Yes (sinus debridement)	Died
Meshram et al. 2021 [50]	India	Amphotericin B	Yes (maxillectomy)	Died
Mishra et al. 2021 [68]	India	Amphotericin B	Yes ((all patients (mixed or any single surgery): functional endoscopic sinus surgery, endoscopic maxillectomy, local debridement)	Lived (50%), Died (40%), Lost to follow-up (10%)
Moorthy et al. 2021 [51]	India	Amphotericin B (5 mg/kg/day)	Yes (FESS (n = 17), maxillectomy(n = 11), exenteration (n = 11))	Died (35.29%)
Nehara et al. 2021 [52]	India	Amphotericin B (LAmB 5 mg/kg/day), posaconazole	No	Lived (60%), Died (40%)
Pakdel et al. 2021 [53]	Iran	Amphotericin B (LAmB 5 mg/kg/day), oral posaconazole	Yes (sinus debridement (n = 12); orbital externation (n = 5); palatal debridement (n = 2))	Lived (53%), Died (47%)
Pasero et al. 2020 [54]	Italy	Amphotericin B/isavuconazole	No	Died
Pauli et al. 2021 [55]	Brazil	Amphotericin B	Yes (debridement)	Lived
Placik et al. 2020 [56]	USA	Amphotericin B	Yes (resection)	Died
Rabagliati et al. 2021 [57]	Chile	Amphotericin B (LAmB)	No	Died
Rao et al. 2021 [58]	India	Amphotericin B (LAmB)	Yes (endoscopic sinus surgery)	NR
Ravani et al. 2021 [59]	India	Amphotericin B (LAmB 5 mg/kg/day)	Yes (sinus debridement; n = 18)	Lived (94%), Died (6%)
Revannavar et al. 2021 [60]	India	Amphotericin B	Yes (endoscopic sinus surgery)	Lived
Saldanha et al. 2021 [61]	India	Amphotericin B (25 mg/day)	Yes (endoscopic sinus surgery)	Lived
Sarkar et al. 2021 [62]	India	Amphotericin B	Yes (maxillectomy (n = 3), debridement (n = 1))	Died
Satish et al. 2021 [69]	India	Amphotericin B	Yes (all patients: surgical debridement)	No data
Sen et al. 2021 [63]	India	Amphotericin B (LAmB)+ voriconazole/posaconazole	Yes (exenteration (n = 2), sinus debridement (n = 3))	Lived
Veisi et al. 2021 [64]	Iran	Amphotericin B (4 mg/kg/day)	Yes (surgical debridement)	Died
		Amphotericin B (3 mg/kg/day)	Yes (endoscopic sinus surgery)	Lived
Waizel-Haiat et al. 2021 [65]	Mexico	Amphotericin B	No	Died
Werthman-Ehrenreich et al. 2021 [66]	USA	Amphotericin B	Yes (sinus debridement)	Died
Zurl et al. 2021 [67]	Austria	No	No	Died

AMB: amphotericin B; IV: intravenous; LAmB: liposomal amphotericin B; NR: not reported; UK: United Kingdom; USA: United States of America. * No separate outcomes were reported for mucormycosis (n = 28).

## Data Availability

The data that support the findings of this study are available from the corresponding author (S.H. or M.K.) upon reasonable request.

## Supplementary Materials

The following are available online at https://www.mdpi.com/article/10.3390/ijerph181910340/s1, Table S1: PRISMA-ScR checklist, Table S2: Search strategy, Table S3: List of excluded articles.

## Appendix A. MEDLINE^®^ ALL <1946 to 8 June 2021> (Ovid)

**#**	**Search String**	**No. of Results**
1	exp Coronavirus/	77,269
2	exp Coronavirus Infections/	94,303
3	(coronavirus* or corona virus* or OC43 or NL63 or 229E or HKU1 or HCoV* or ncov* or covid* or sars-cov* or sarscov* or Sars-coronavirus* or Severe Acute Respiratory Syndrome Coronavirus* or “Kawasaki like paediatric inflammatory multisystem syndrome” or “Kawasaki like pediatric inflammatory multisystem syndrome” or “PIMS-TS” or “Kawa-COVID-19” or “MIS-C” or “multisystem inflammatory syndrome in children” or pediatric multisystem inflammatory disease).mp.	159,987
4	(or/1–3) and ((20191* or 202*).dp. or 20190101:20301231.(ep).) (147001)	147,001
5	4 not (SARS or SARS-CoV or MERS or MERS-CoV or Middle East respiratory syndrome or camel * or dromedary* or equine or coronary or coronal or covidence* or covidien or influenza virus or HIV or bovine or calves or TGEV or feline or porcine or BCoV or PED or PEDV or PDCoV or FIPV or FCoV or SADS-CoV or canine or CCov or zoonotic or avian influenza or H1N1 or H5N1 or H5N6 or IBV or murine corona*).mp.	54,231
6	((pneumonia or covid* or coronavirus* or corona virus* or ncov* or 2019-ncov or sars*).mp. or exp pneumonia/) and Wuhan.mp.	5278
7	(2019-ncov or ncov19 or ncov-19 or 2019-novel CoV or sars-cov2 or sars-cov-2 or sarscov2 or sarscov-2 or SARS-2-nCoV or SARS-2-Cov or SARS-COV-19 or Sars-coronavirus2 or Sars-coronavirus-2 or SARS 2 coronavirus* or Severe Acute Respiratory Syndrome-CoV-2 or SARS-like coronavirus* or coronavirus-19 or covid19 or covid-19 or covid 2019 or ((novel or new or nouveau) adj2 (CoV or nCoV or covid or coronavirus* or corona virus or Pandemi*2)) or ((covid or covid19 or covid-19 or SARS-CoV-2) and pandemic*2) or (coronavirus* and pneumonia)).mp.	144,923
8	(COVID-19 or SARS-CoV-2).rx,px,ox,rn. or (COVID-19 or COVID-19 serotherapy or ORF7b protein, SARS-CoV-2 or ORF6 protein, SARS-CoV-2 or ORF8 protein, SARS-CoV-2 or pediatric multisystem inflammatory disease, COVID-19 related or envelope protein, SARS-CoV-2 or ORF7a protein, SARS-CoV-2 or spike protein, SARS-CoV-2 or ORF3a protein, SARS-CoV-2 or COVID-19 drug treatment or severe acute respiratory syndrome coronavirus 2 or membrane protein, SARS-CoV-2 or ORF1ab polyprotein, SARS-CoV-2 or nucleocapsid protein, Coronavirus or COVID-19 vaccine or COVID-19 diagnostic testing).os,ps,rn,rs.	8460
9	(“32185863” or “32172715” or “32227595” or “32140676” or “32246156” or “32267941” or “32176889” or “32169616” or “32265186” or “32253187” or “32152148” or “32053580” or “32179788” or “32213260” or “32205350” or “32188729” or “32152361” or “32277065” or “32088947” or “32240583” or “31917786” or “32127714” or “32047315” or “32020111” or “32240632” or “32243118” or “32267344” or “32239781” or “32396977” or “32402130” or “32243299” or “32807526” or “32344395” or “32403202” or “32389714” or “32416016” or “32405099” or “32976849” or “32685966” or “33221888” or “32379271” or “32188728” or “32221976” or “32417321” or “32489438” or “32332959” or “32943452” or “32807525” or “32826274” or “32898560” or “32293023” or “33159926” or “32919952” or “32835716” or “32619499” or “32663524” or “32392627” or “32392625” or “33037657” or “32777045” or “32521569” or “32492200” or “32930765” or “33075143” or “32237249” or “32683439” or “32495994” or “32344447” or “32896006” or “32240549” or “32438448” or “32425477” or “32951095” or “32274794” or “32750178” or “32463935” or “32428286” or “32491981” or “32930748” or “32119409” or “32432657” or “33003176” or “32459319” or “32822920” or “32878290” or “32270498” or “32250493” or “32512243” or “32837399” or “32426074” or “32199942” or “32839969” or “32639522” or “33073717” or “32502134” or “32334003” or “32510470” or “32819741” or “32309248” or “32243951” or “32378772” or “32835361” or “32962779” or “32916324” or “32785973” or “32272221” or “32299207” or “33044515” or “33134955” or “32970917” or “32407438” or “32513790” or “32439468” or “33063036” or “33077677” or “32406056” or “32716821” or “32588590” or “32239757” or “32829902” or “32807521” or “32379350” or “33125767” or “32829731” or “32988821” or “32780977” or “32648633” or “32829907” or “32330635” or “32692998” or “33013067” or “33010706” or “32502292” or “32780969” or “32998780” or “32754731” or “32639607” or “32233030” or “32953429” or “32246897” or “32955802” or “32425490” or “32418270” or “32445255” or “32775945” or “32775948” or “32775953” or “32407043”).ui.	152
10	or/5–9	147,637
11	10 and 20191201:20301231.(dt).	145,485
12	exp Zygomycosis/	4474
13	mucormycos#s.mp.	5053
14	Mucormycose.mp.	98
15	mucoromycos#s.mp.	6
16	zygomycos#s.mp.	1414
17	(black fungus or black fungi).mp.	192
18	exp Mucorales/	6616
19	Mucorales.mp.	192
20	mucoralean.mp.	70
21	Absidia.mp.	562
22	Cunninghamella.mp.	768
23	Mortierella.mp.	751
24	Mucor.mp.	3382
25	Apophysomyces.mp.	147
26	Saksenaea.mp.	102
27	Rhizopus.mp.	4211
28	Rhizomucor.mp.	691
29	Lichtheimia.mp.	191
30	Cokeromyces.mp.	24
31	Actinomucor.mp.	58
32	Syncephalastrum.mp.	163
33	12 or 13 or 14 or 15 or 16 or 17 or 18 or 19 or 20 or 21 or 22 or 23 or 24 or 25 or 26 or 27 or 28 or 29 or 30 or 31 or 32	14,534
34	11 and 33	57
Search was conducted on 9 June 2021 at 4:25 p.m. (CET).		
