# Supplementary material for: COVID-19-Associated Mucormycosis (CAM): An Updated Evidence Mapping
Source: Int J Environ Res Public Health. 2021 Sep 30;18(19):10340. doi: 10.3390/ijerph181910340 (PMC8508302; doi:10.3390/ijerph181910340)
Supplement: Supplementary file 1 [file ijerph-18-10340-s001.zip › ijerph-1339035-supplementary.pdf]

Supplementary File S1

Preferred Reporting Items for Systematic reviews and Meta-Analyses extension for Scoping Reviews (PRISMA-ScR) Checklist

SECTION	ITEM	PRISMA-ScR CHECKLIST ITEM	REPORTED ON PAGE #
**TITLE**			
Title	1	Identify the report as a scoping review.	1
**ABSTRACT**			
Structured summary	2	Provide a structured summary that includes (as applicable): background, objectives, eligibility criteria, sources of evidence, charting methods, results, and conclusions that relate to the review questions and objectives.	2
**INTRODUCTION**			
Rationale	3	Describe the rationale for the review in the context of what is already known. Explain why the review questions/objectives lend themselves to a scoping review approach.	2-3
Objectives	4	Provide an explicit statement of the questions and objectives being addressed with reference to their key elements (e.g., population or participants, concepts, and context) or other relevant key elements used to conceptualize the review questions and/or objectives.	3
**METHODS**			
Protocol and registration	5	Indicate whether a review protocol exists; state if and where it can be accessed (e.g., a Web address); and if available, provide registration information, including the registration number.	3
Eligibility criteria	6	Specify characteristics of the sources of evidence used as eligibility criteria (e.g., years considered, language, and publication status), and provide a rationale.	3
Information sources*	7	Describe all information sources in the search (e.g., databases with dates of coverage and contact with authors to identify additional sources), as well as the date the most recent search was executed.	3
Search	8	Present the full electronic search strategy for at least 1 database, including any limits used, such that it could be repeated.	Supplement
Selection of sources of evidence†	9	State the process for selecting sources of evidence (i.e., screening and eligibility) included in the scoping review.	3-4
Data charting process‡	10	Describe the methods of charting data from the included sources of evidence (e.g., calibrated forms or forms that have been tested by the team before their use, and whether data charting was done	4

SECTION	ITEM	PRISMA-ScR CHECKLIST ITEM	REPORTED ON PAGE #
		independently or in duplicate) and any processes for obtaining and confirming data from investigators.	
Data items	11	List and define all variables for which data were sought and any assumptions and simplifications made.	4
Critical appraisal of individual sources of evidence§	12	If done, provide a rationale for conducting a critical appraisal of included sources of evidence; describe the methods used and how this information was used in any data synthesis (if appropriate).	NA
Synthesis of results	13	Describe the methods of handling and summarizing the data that were charted.	4
**RESULTS**			
Selection of sources of evidence	14	Give numbers of sources of evidence screened, assessed for eligibility, and included in the review, with reasons for exclusions at each stage, ideally using a flow diagram.	4
Characteristics of sources of evidence	15	For each source of evidence, present characteristics for which data were charted and provide the citations.	5
Critical appraisal within sources of evidence	16	If done, present data on critical appraisal of included sources of evidence (see item 12).	NA
Results of individual sources of evidence	17	For each included source of evidence, present the relevant data that were charted that relate to the review questions and objectives.	5
Synthesis of results	18	Summarize and/or present the charting results as they relate to the review questions and objectives.	5, Table 1, Table 2
**DISCUSSION**			
Summary of evidence	19	Summarize the main results (including an overview of concepts, themes, and types of evidence available), link to the review questions and objectives, and consider the relevance to key groups.	6
Limitations	20	Discuss the limitations of the scoping review process.	7
Conclusions	21	Provide a general interpretation of the results with respect to the review questions and objectives, as well as potential implications and/or next steps.	7
**FUNDING**			
Funding	22	Describe sources of funding for the included sources of evidence, as well as sources of funding for the scoping review. Describe the role of the funders of the scoping review.	7

JBI = Joanna Briggs Institute; PRISMA-ScR = Preferred Reporting Items for Systematic reviews and Meta-Analyses extension for Scoping Reviews.

* Where *sources of evidence* (see second footnote) are compiled from, such as bibliographic databases, social media platforms, and Web sites.

† A more inclusive/heterogeneous term used to account for the different types of evidence or data sources (e.g., quantitative and/or qualitative research, expert opinion, and policy documents) that may be eligible in a scoping review as opposed to only studies. This is not to be confused with *information sources* (see first footnote).
‡ The frameworks by Arksey and O'Malley (6) and Levac and colleagues (7) and the JBI guidance (4, 5) refer to the process of data extraction in a scoping review as data charting.
§ The process of systematically examining research evidence to assess its validity, results, and relevance before using it to inform a decision. This term is used for items 12 and 19 instead of "risk of bias" (which is more applicable to systematic reviews of interventions) to include and acknowledge the various sources of evidence that may be used in a scoping review (e.g., quantitative and/or qualitative research, expert opinion, and policy document).

From: Tricco AC, Lillie E, Zarin W, O'Brien KK, Colquhoun H, Levac D, et al. PRISMA Extension for Scoping Reviews (PRISMAScR): Checklist and Explanation. Ann Intern Med. 2018;169:467–473. doi: 10.7326/M18-0850.

Supplementary File S2

MEDLINE(R) ALL <1946 to June 08, 2021> (Ovid)

Search was conducted on 9th June 2021 at 4:25 pm (CET).

#	search string	# of results
**1**	exp Coronavirus/	77269
**2**	exp Coronavirus Infections/	94303
**3**	(coronavirus* or corona virus* or OC43 or NL63 or 229E or HKU1 or HCoV* or ncov* or covid* or sars-cov* or sarscov* or Sars-coronavirus* or Severe Acute Respiratory Syndrome Coronavirus* or "Kawasaki like paediatric inflammatory multisystem syndrome" or "Kawasaki like pediatric inflammatory multisystem syndrome" or "PIMS-TS" or "Kawa-COVID-19" or "MIS-C" or "multisystem inflammatory syndrome in children" or pediatric multisystem inflammatory disease).mp.	159987
**4**	(or/1-3) and ((20191* or 202*).dp. or 20190101:20301231.(ep).) (147001)	147001
**5**	4 not (SARS or SARS-CoV or MERS or MERS-CoV or Middle East respiratory syndrome or camel* or dromedar* or equine or coronary or coronal or covidence* or covidien or influenza virus or HIV or bovine or calves or TGEV or feline or porcine or BCoV or PED or PEDV or PDCoV or FIPV or FCoV or SADS-CoV or canine or CCov or zoonotic or avian influenza or H1N1 or H5N1 or H5N6 or IBV or murine corona*).mp.	54231
**6**	((pneumonia or covid* or coronavirus* or corona virus* or ncov* or 2019-ncov or sars*).mp. or exp pneumonia/) and Wuhan.mp.	5278
**7**	(2019-ncov or ncov19 or ncov-19 or 2019-novel CoV or sars-cov2 or sars-cov-2 or sarscov2 or sarscov-2 or SARS-2-nCoV or SARS-2-Cov or SARS-COV-19 or Sars-coronavirus2 or Sars-coronavirus-2 or SARS 2 coronavirus* or Severe Acute Respiratory Syndrome-CoV-2 or SARS-like coronavirus* or coronavirus-19 or covid19 or covid-19 or covid 2019 or ((novel or new or nouveau) adj2 (CoV or nCoV or covid or coronavirus* or corona virus or Pandemi*2)) or ((covid or covid19 or covid-19 or SARS-CoV-2) and pandemic*2) or (coronavirus* and pneumonia)).mp.	144923
**8**	(COVID-19 or SARS-CoV-2).rx,px,ox,rn. or (COVID-19 or COVID-19 serotherapy or ORF7b protein, SARS-CoV-2 or ORF6 protein, SARS-CoV-2 or ORF8 protein, SARS-CoV-2 or pediatric multisystem inflammatory	8460

	disease, COVID-19 related or envelope protein, SARS-CoV-2 or ORF7a protein, SARS-CoV-2 or spike protein, SARS-CoV-2 or ORF3a protein, SARS-CoV-2 or COVID-19 drug treatment or severe acute respiratory syndrome coronavirus 2 or membrane protein, SARS-CoV-2 or ORF1ab polyprotein, SARS-CoV-2 or nucleocapsid protein, Coronavirus or COVID-19 vaccine or COVID-19 diagnostic testing).os,ps,rn,rs.	
**9**	("32185863" or "32172715" or "32227595" or "32140676" or "32246156" or "32267941" or "32176889" or "32169616" or "32265186" or "32253187" or "32152148" or "32053580" or "32179788" or "32213260" or "32205350" or "32188729" or "32152361" or "32277065" or "32088947" or "32240583" or "31917786" or "32127714" or "32047315" or "32020111" or "32240632" or "32243118" or "32267344" or "32239781" or "32396977" or "32402130" or "32243299" or "32807526" or "32344395" or "32403202" or "32389714" or "32416016" or "32405099" or "32976849" or "32685966" or "33221888" or "32379271" or "32188728" or "32221976" or "32417321" or "32489438" or "32332959" or "32943452" or "32807525" or "32826274" or "32898560" or "32293023" or "33159926" or "32919952" or "32835716" or "32619499" or "32663524" or "32392627" or "32392625" or "33037657" or "32777045" or "32521569" or "32492200" or "32930765" or "33075143" or "32237249" or "32683439" or "32495994" or "32344447" or "32896006" or "32240549" or "32438448" or "32425477" or "32951095" or "32274794" or "32750178" or "32463935" or "32428286" or "32491981" or "32930748" or "32119409" or "32432657" or "33003176" or "32459319" or "32822920" or "32878290" or "32270498" or "32250493" or "32512243" or "32837399" or "32426074" or "32199942" or "32839969" or "32639522" or "33073717" or "32502134" or "32334003" or "32510470" or "32819741" or "32309248" or "32243951" or "32378772" or "32835361" or "32962779" or "32916324" or "32785973" or "32272221" or "32299207" or "33044515" or "33134955" or "32970917" or "32407438" or "32513790" or "32439468" or "33063036" or "33077677" or "32406056" or "32716821" or "32588590" or "32239757" or "32829902" or "32807521" or "32379350" or "33125767" or "32829731" or "32988821" or "32780977" or "32648633" or "32829907" or "32330635" or "32692998" or "33013067" or "33010706" or "32502292" or "32780969" or "32998780" or "32754731" or "32639607" or "32233030" or "32953429" or "32246897" or "32955802" or "32425490" or "32418270" or "32445255" or "32775945" or "32775948" or "32775953" or "32407043").ui.	152
**10**	or/5-9	147637
**11**	10 and 20191201:20301231.(dt).	145485

**12**	exp Zygomycosis/	4474
**13**	mucormycos#s.mp.	5053
**14**	Mucormycose.mp.	98
**15**	mucoromycos#s.mp.	6
**16**	zygomycos#s.mp.	1414
**17**	(black fungus or black fungi).mp.	192
**18**	exp Mucorales/	6616
**19**	Mucorales.mp.	192
**20**	mucoralean.mp.	70
**21**	Absidia.mp.	562
**22**	Cunninghamella.mp.	768
**23**	Mortierella.mp.	751
**24**	Mucor.mp.	3382
**25**	Apophysomyces.mp.	147
**26**	Saksenaea.mp.	102
**27**	Rhizopus.mp.	4211
**28**	Rhizomucor.mp.	691
**29**	Lichtheimia.mp.	191
**30**	Cokeromyces.mp.	24
**31**	Actinomucor.mp.	58
**32**	Syncephalastrum.mp.	163
**33**	12 or 13 or 14 or 15 or 16 or 17 or 18 or 19 or 20 or 21 or 22 or 23 or 24 or 25 or 26 or 27 or 28 or 29 or 30 or 31 or 32	14534

**34**	11 and 33	57

Embase <1974 to 2021 June 08> (Ovid)

Search was conducted on 9th June 2021 at 4:30 pm (CET).

#	search string	# of results
**1**	exp Coronavirus/	52407
**2**	exp Coronavirus Infections/	138125
**3**	(coronavirus* or corona virus* or OC43 or NL63 or 229E or HKU1 or HCoV* or ncov* or covid* or sars-cov* or sarscov* or Sars-coronavirus* or Severe Acute Respiratory Syndrome Coronavirus* or "Kawasaki like paediatric inflammatory multisystem syndrome" or "Kawasaki like pediatric inflammatory multisystem syndrome" or "PIMS-TS" or "Kawa-COVID-19" or "MIS-C" or "multisystem inflammatory syndrome in children" or pediatric multisystem inflammatory disease).mp.	174476
**4**	(or/1-3) and 20190101:20301231.(dc).	153718
**5**	4 not (SARS or SARS-CoV or MERS or MERS-CoV or Middle East respiratory syndrome or camel* or dromedar* or equine or coronary or coronal or covidence* or covidien or influenza virus or HIV or bovine or calves or TGEV or feline or porcine or BCoV or PED or PEDV or PDCoV or FIPV or FCoV or SADS-CoV or canine or CCov or zoonotic or avian influenza or H1N1 or H5N1 or H5N6 or IBV or murine corona*).mp.	95190
**6**	((pneumonia or covid* or coronavirus* or corona virus* or ncov* or 2019-ncov or sars*).mp. or exp pneumonia/) and Wuhan.mp.	5450
**7**	(coronavirus disease 2019 or 2019-ncov or 2019nCoV or ncov2019 or ncov19 or ncov-19 or HCoV-19 or 2019-novel CoV or severe acute respiratory syndrome coronavirus 2 or sars2 or sars 2 or sars-cov2 or sars-cov-2 or sarscov2 or sarscov-2 or Sars-coronavirus2 or Sars-coronavirus-2 or SARS-CoV-19 or SARS-2-nCoV or SARS-2-Cov or SARS 2 coronavirus* or Severe Acute Respiratory Syndrome-CoV-2 or SARS-like coronavirus* or coronavirus-19 or covid19 or covid-19 or covid 2019 or ((novel or new or nouveau) adj2 (CoV or nCoV or covid or coronavirus* or corona virus or Pandemi*2)) or ((covid or covid19 or covid-19 or SARS-CoV-2) and pandemic*2) or (coronavirus* and pneumonia)).mp.	150869
**8**	(coronavirus disease 2019 or severe acute respiratory syndrome coronavirus 2).sh,dj.	123101

**9**	("630575119" or "630830186" or "630941329" or "631043694" or "631260659" or "631272428" or "631272880" or "631286076" or "631290163" or "631308782" or "631324397" or "631352500" or "631416440" or "631431802" or "631452886" or "631456079" or "631457551" or "631462438" or "631462876" or "631465538" or "631465685" or "631469310" or "2004499662" or "2004505338" or "2005280837" or "2005387675" or "2005408544" or "2005484987" or "2005549151").an.	16
**10**	(or/6-9) and 20191201:20301231.(dc).	147750
**11**	5 or 10	149742
**12**	zygomycosis/ or mucormycosis/	7409
**13**	mucormycos#s.mp.	7396
**14**	Mucormycose.mp.	112
**15**	mucoromycos#s.mp.	7
**16**	zygomycos#s.mp.	1689
**17**	(black fungus or black fungi).mp.	206
**18**	exp Mucorales/	11291
**19**	Mucorales.mp.	1863
**20**	mucoralean.mp.	88
**21**	Absidia.mp.	855
**22**	Cunninghamella.mp.	1126
**23**	Mortierella.mp.	882
**24**	Mucor.mp.	5017
**25**	Apophysomyces.mp.	204
**26**	Saksenaea.mp.	135
**27**	Rhizopus.mp.	6384

**28**	Rhizomucor.mp.	1207
**29**	Lichtheimia.mp.	433
**30**	Cokeromyces.mp.	29
**31**	Actinomucor.mp.	71
**32**	Syncephalastrum.mp.	285
**33**	12 or 13 or 14 or 15 or 16 or 17 or 18 or 19 or 20 or 21 or 22 or 23 or 24 or 25 or 26 or 27 or 28 or 29 or 30 or 31 or 32	19842
**34**	11 and 33	52

Cochrane COVID-19 Study Register

Search was conducted on 9th June 2021 at 4:45 pm (CET).

#	**search string**	**# of results**
**1**	mucormycosis OR mucormycoses OR mucormycose OR mucoromycosis OR mucoromycoses OR zygomycosis OR zygomycoses OR "black fungus" OR "black fungi" OR Mucorales OR mucoralean OR Absidia OR Cunninghamella OR Mortierella OR Mucor OR Apophysomyces OR Saksenaea OR Rhizopus OR Rhizomucor OR Lichtheimia OR Cokeromyces OR Actinomucor OR Syncephalastrum	37

WHO COVID-19 database

Search was conducted on 9th June 2021 at 5:10 pm (CET).

#	**search string**	**# of results**
**1**	MH:C01.150.703.980* OR MH: B01.300.300.500* OR mucormycosis OR mucormycoses OR mucormycose OR mucoromycosis OR mucoromycoses OR zygomycosis OR zygomycoses OR "black fungus" OR "black fungi" OR Mucorales OR mucoralean OR Absidia OR Cunninghamella OR Mortierella OR Mucor OR Apophysomyces OR Saksenaea OR Rhizopus OR Rhizomucor OR Lichtheimia OR Cokeromyces OR Actinomucor OR Syncephalastrum	59

Supplementary File S3

List of articles excluded during full-text screening with reason

Study author, year	Title	Reason for exclusion
Ahmad et al. 2020 [1]	Imaging spectrum of pulmonary infections in renal transplant patients	Review article
Ahmadikia et al. 2021 [2]	The double-edged sword of systemic corticosteroid therapy in viral pneumonia: A case report and comparative review of influenza-associated mucormycosis versus COVID-19 associated mucormycosis	Population not of interest (non-COVID patients)
Aljehani et al. 2021 [3]	A Case Report of Complete Resolution of Auricular Mucormycosis in an 18-Month-Old Diabetic Child	Population not of interest (non-COVID patients)
Clinical trial (NCT04368221) [4]	Characterization of Fungal Infections in COVID-19 Infected and Mechanically Ventilated Patients in ICU	Protocol
Fekkar et al. 2021 [5]	Occurrence of invasive pulmonary fungal infections in patients with severe COVID-19 admitted to the ICU	No confirmed outcomes (mixed fungal infections)
Gonzalez-Calatayud et al. 2020 [6]	Observational study of the suspected or confirmed cases of sars COV-2 infection needing emergency surgical intervention during the first months of the pandemic in a third level hospital: Case series	No confirmed mucormycosis as per our inclusion criteria
Sharma et al. 2021 [7]	Post coronavirus disease mucormycosis: a deadly addition to the pandemic spectrum	No confirmed mucormycosis as per our inclusion criteria

References:

1. Ahmad Z, Bagchi S, Naranje P, Agarwal SK, Das CJ. Imaging spectrum of pulmonary infections in renal transplant patients. The Indian Journal of Radiology & Imaging. 2020;30(3):273.
2. Ahmadikia K, Hashemi SJ, Khodavaisy S, Getso MI, Alijani N, Badali H, et al. The double-edged sword of systemic corticosteroid therapy in viral pneumonia: A case report and comparative review of influenza-associated mucormycosis versus COVID-19 associated mucormycosis. Mycoses. 2021.

3. Aljehani Aljehani M, Alahmadi H, Alshamani M. A Case Report of Complete Resolution of Auricular Mucormycosis in an 18-Month-Old Diabetic Child. Case Reports in Otolaryngology. 2021.
4. Characterization of Fungal Infections in COVID-19 Infected and Mechanically Ventilated Patients in ICU. Available at: https://clinicaltrials.gov/ct2/show/NCT04368221.
5. Fekkar A, Lampros A, Mayaux J, Poignon C, Demeret S, Constantin JM, et al. Occurrence of invasive pulmonary fungal infections in patients with severe COVID-19 admitted to the ICU. American Journal of Respiratory and Critical Care Medicine. 2021;203(3):307-17.
6. González-Calatayud M, Vargas-Ábrego B, Gutiérrez-Uvalle GE, López-Romero SC, González-Pérez LG, Carranco-Martínez JA, Raful-Zacarías-Ezzat J, Gracida-Mancilla NI. Observational study of the suspected or confirmed cases of sars COV-2 infection needing emergency surgical intervention during the first months of the pandemic in a third level hospital: Case series. Annals of Medicine and Surgery. 2020;60:149-54.
7. Sharma S, Grover M, Bhargava S, Samdani S, Kataria T. Post coronavirus disease mucormycosis: a deadly addition to the pandemic spectrum. The Journal of Laryngology & Otology. 2021;135(5):442-7.
